# Optimizing Waste Heat Conversion: Integrating Phase-Change Material Heatsinks and Wind Speed Dynamics to Enhance Flexible Thermoelectric Generator Efficiency

**DOI:** 10.3390/ma17020420

**Published:** 2024-01-14

**Authors:** Phanathagorn Egypt, Rachsak Sakdanuphab, Aparporn Sakulkalavek, Bhanupol Klongratog, Nuttakrit Somdock

**Affiliations:** 1 Department of Physics, School of Science, King Mongkut’s Institute of Technology Ladkrabang, Bangkok 10520, Thailand; 64605082@kmitl.ac.th (P.E.); aparporn.sa@kmitl.ac.th (A.S.); nuttakrit.so@kmitl.ac.th (N.S.); 2 College of Advanced Manufacturing Innovation, King Mongkut’s Institute of Technology Ladkrabang, Ladkrabang, Bangkok 10520, Thailand; rachsak.sa@kmitl.ac.th; 3 Electronic and Optoelectronic Device Research Unit, Faculty of Science, King Mongkut’s Institute of Technology Ladkrabang, Bangkok 10520, Thailand

**Keywords:** flexible thermoelectric generators, phase-change materials, waste heat conversion, power density, energy harvesting

## Abstract

Flexible thermoelectric generators (FTEGs) have garnered significant attention for their potential in harnessing waste heat energy from various sources. To optimize their efficiency, FTEGs require efficient and adaptable heatsinks. In this study, we propose a cost-effective solution by integrating phase-change materials into FTEG heatsinks. We developed and tested three flexible phase-change material thicknesses (4 mm, 7 mm, and 10 mm), focusing on preventing leaks during operation. Additionally, we investigated the impact of wind speed on the output performance of FTEGs with a flexible phase-change material heatsink. The results indicate that the appropriate flexible phase-change material thickness, when integrated with considerations for wind speed, demonstrates remarkable heat-absorbing capabilities at phase-change temperatures. This integration enables substantial temperature differentials across the FTEG modules. Specifically, the FTEG equipped with a 10 mm thick flexible phase-change material heatsink achieved a power density more than four times higher when the wind speed was at 1 m/s compared to no wind speed. This outcome suggests that integrating phase-change material heatsinks with relatively low wind speeds can significantly enhance flexible thermoelectric generator efficiency. Finally, we present a practical application wherein the FTEG, integrated with the flexible phase-change material heatsink, efficiently converts waste heat from a circular hot pipe into electricity, serving as a viable power source for smartphone devices. This work opens exciting possibilities for the future integration of flexible thermoelectric modules with flexible phase-change material heatsinks, offering a promising avenue for converting thermal waste heat into usable electricity.

## 1. Introduction

Low-level heat, typically below 100 °C, represents a plentiful yet frequently squandered energy resource [1,2,3]. A significant portion of the energy used in worldwide industrial production, approximately 72%, is wasted in the form of low-grade heat [1,4]. Notably, the natural sources of low-grade heat, such as geothermal activity and solar radiation, surpass even the total annual energy consumption [5,6,7]. Therefore, capturing and utilizing this immense amount of energy offers great potential for creating a more sustainable society [8]. Thermoelectric generators (TEGs) have demonstrated the ability to directly convert heat into electricity via temperature differences [9,10]. In recent years, multiple methods have been explored to develop flexible thermoelectric generators (FTEGs) [2,5,6,7,8,11,12]. These FTEGs exhibit great potential due to their impressive thermoelectric capabilities and flexibility, making them ideal for harnessing wasted heat from various sources, even those with curved or uneven surfaces [12,13,14]. In recent studies conducted by Kim et al. [15] and subsequent research [16,17,18], FTEGs with exceptional performance have been developed. However, the power output relies not solely on the efficiency of FTEGs, but also on an effective heatsink system. Conventional metal heatsinks are unsuitable for use with FTEGs due to their inflexibility and bulky nature. Finding a suitable heatsink that offers both high cooling performance and flexibility is a challenging task.

In recent developments, innovative flexible heatsinks have emerged as solutions to address these issues. These heatsinks utilize materials such as Super Absorbent Polymer (SAP) [19] and a combination of solid-state silica gel with hydrogel [20,21]. They harness heat from water evaporation and its sensible heat, delivering impressive cooling performance, excellent portability, and extended operational durations. However, it is important to note that these flexible heatsinks require periodic water replenishment to achieve semi-permanent functionality and cannot operate autonomously [22].

Hence, a flexible heatsink structure based on a phase-change material (PCM) has been discussed in recent studies. PCM can absorb or release a substantial amount of heat, known as latent heat, during its phase transition [22,23]. Nevertheless, the primary concern when employing PCM lies in the possibility of melting and leakage when exposed to certain low-level heat sources with temperatures ranging from 60 to 100 °C. Additionally, natural wind convection is a suitable option to combine with a flexible phase-change material heatsink because wind energy arises in real-world situations due to differences in the atmospheric pressure, and it can operate autonomously.

This study introduces a flexible heatsink design, incorporating a phase-change material (RT42), aimed at preventing leaks during operation. Three thicknesses (4 mm, 7 mm, and 10 mm) of flexible phase-change material were developed and employed to regulate the heat dissipation of the FTEG. In this work, flexible phase-change material heatsinks with a maximum thickness of 10 mm were fabricated. Thicknesses exceeding 10 mm were not produced due to their inability to bend. In addition, the effect of wind speed on the output performance of the FTEG with a flexible phase-change material heatsink was investigated. Finally, a practical application employing the fabricated FTEG has been successfully conducted, converting the heat from a hot pipe into electricity and demonstrating its potential as an electrical power source for a smartphone device.

## 2. Experimental Section

### 2.1. Flexible Thermoelectric Design and Fabrication

**Figure 1:**
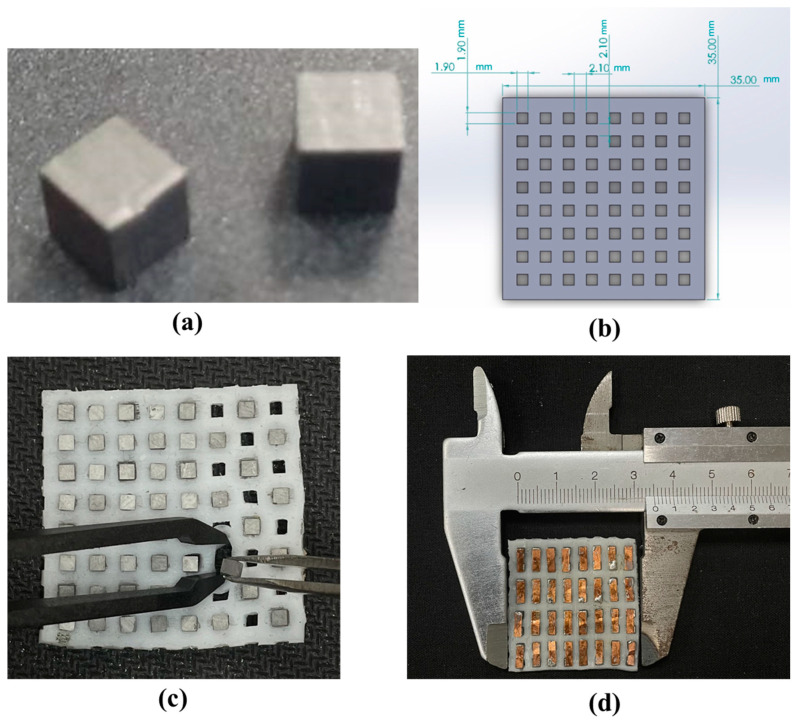
Flexible thermoelectric module manufacturing process. (**a**) Thermoelectric components sized at 2 × 2 × 2 mm^3^. (**b**) A silicone rubber sheet template with 8 rows and 8 columns; each hole measures 1.9 × 1.9 mm^2^, with the thermoelectric material evenly spaced at 2 mm intervals. (**c**) Embedding the thermoelectric components within the patterned silicone rubber. (**d**) Prototype of a 3.5 × 3.5 cm^2^ flexible thermoelectric module.

A flexible thermoelectric generator (FTEG) module measuring 3.5 × 3.5 cm^2^ was created. This module was constructed using p-type and n-type thermoelectric legs made of bismuth telluride materials provided by a commercial supplier (Wuhan Xinrong New Materials Co., Ltd., Wuhan, China). The legs were small, measuring 2 × 2 × 2 mm^3^, and had Ni (4 µm) (Siam Jin Charoen Enterprise Co., Ltd., Samut Prakan, Thailand) contacts as transition layers on both sides. They were arranged in an 8 × 8 array with a 2 mm gap between them, resulting in 64 pairs of p-type and n-type elements. To enhance insulation and flexibility, the legs were enclosed in silicone rubber (Fantastic Triumph Co., Ltd., Bangkok, Thailand). An automated dispensing machine placed a dot of Sn_0.955_Ag_0.038_Cu_0.007_ solder paste (Chip Quik, NY, USA), with a diameter of 0.3 mm, onto the patterned electrodes. A copper electrode with a thickness of 0.1 mm was used to connect the p-type and n-type thermoelectric elements. The fabrication process is illustrated in Figure 1. 

### 2.2. Flexible Heatsink Fabrication

**Figure 2:**
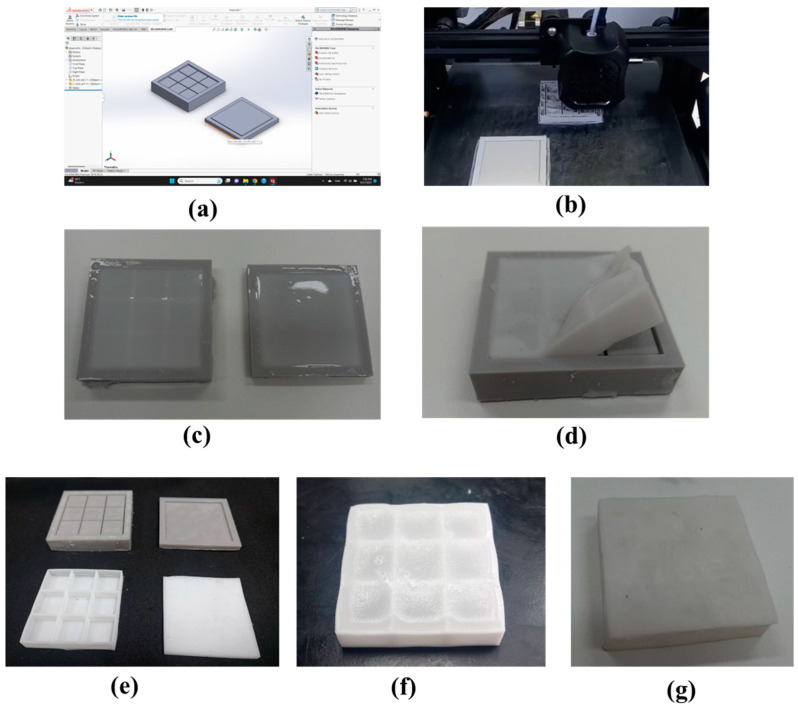
Manufacturing process of flexible phase-change material for heatsink application.

We developed and constructed a flexible phase-change material heatsink to enhance the power generation performance of FTEGs while ensuring leak prevention during operation. The detailed process is illustrated in Figure 2, and a flexible phase-change material heatsink fabrication process is outlined below.

First, we designed a silicone mold using SolidWorks software 2021, which consists of two parts: the structural frame and the top cover (Figure 2a). The structural frame measures 50 × 50 mm^2^ and is divided into nine equal compartments, each measuring 15.3 × 15.3 mm^2^ with 1 mm spacing between them. Each of the nine inner compartments has a 1 mm depth from the outer edges. Next, we 3D-printed the silicone mold, creating three different thicknesses: 4 mm, 7 mm, and 10 mm (Figure 2b). Then, we mixed liquid silicone (Fantastic Triumph Co., Ltd., Bangkok, Thailand) and a catalyst (Fantastic Triumph Co., Ltd., Bangkok, Thailand) at a ratio of 25 g of silicone to 6 drops of catalyst. This mixture was poured into the mold, and the silicone was left to solidify for approximately 2 days (Figure 2c). Once the silicone had hardened, we removed it from the mold (Figure 2d), obtaining both the frame and top cover for the flexible heat dissipation unit (Figure 2e).

The RT42 (Rubitherm® Technologies GmbH, Berlin, Germany) was selected for this work because it is low-cost and safe to use. The thermophysical properties of RT42 are shown in Table 1.

The phase-change material RT42 was heated at 100 °C for 30 min and poured into the frame. Afterward, we waited for approximately 30 min for the phase-change material to solidify once again (Figure 2f). We then placed the top cover on top and connected both components, sealing around the top cover using liquid silicone (Figure 2g). Finally, we attached the heatsink to the flexible thermoelectric module.

### 2.3. Output Power Measurement Setup

**Figure 3:**
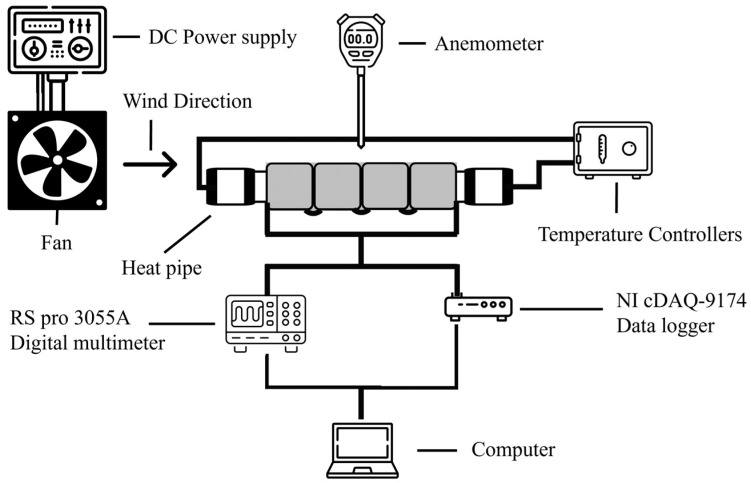
Schematic of the experimental setup for evaluating flexible thermoelectric generator performance.

Figure 3 shows a diagram illustrating the setup used to evaluate the performance of the flexible thermoelectric generator (FTEG) experimentally. The FTEG, which had different thicknesses of a flexible heatsink, was placed on a circular hot pipe. To enhance thermal conductivity, fill air gaps, eliminate thermal interface resistance, improve heat transfer, and compensate for surface imperfections, the flexible thermoelectric device and heatsink were joined together using heat-conducting silicone grease. The temperature of the hot pipe was carefully regulated at 80 °C. During the heating process, the open-circuit voltage (*Voc*) was measured, along with the temperatures on the hot side and cold side of the FTEG. The temperatures of the hot and cold sides were monitored using a K-type thermocouple, and the temperature data were recorded using a National Instruments NI cDAQ-9174 (National Instruments Corporation, Austin, TX, USA) data logger to capture the temperature behavior. Moreover, a fan, controlled by a DC (direct current) power supply, facilitated airflow at particular wind speeds. The wind speed was contingent on the governor current and was measured using an anemometer. The open-circuit voltage (*Voc*) was measured using an RS pro 3055A digital multimeter (RS Group Plc., London, England, UK). The output power was estimated according to Equation (1)

(1) $$P=\frac{V_{OC}^{2}}{4R_{out}}$$

where *R_out_* is the matching load resistance of the FTEG.

## 3. Results and Discussion

### 3.1. Flexible Thermoelectric Module

**Figure 4:**
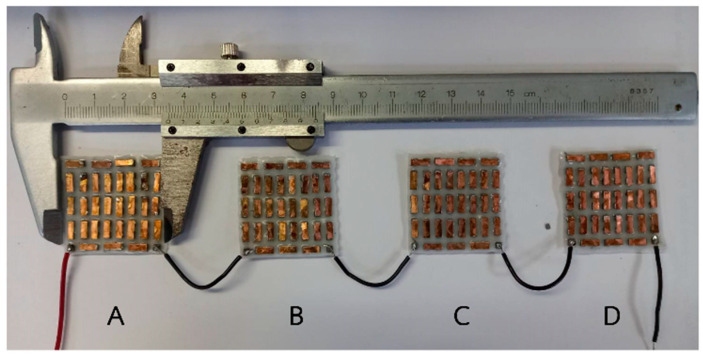
Four prototype flexible thermoelectric modules, each measuring 3.5 × 3.5 cm^2^. (**A**–**D**) refer to the 1st, 2nd, 3rd and 4th flexible thermoelectric modules.

The dimensions of the flexible thermoelectric module were measured using a vernier caliper, as depicted in Figure 4. To determine the electrical resistance of the flexible thermoelectric module, we employed a digital multimeter. The electrical resistance was measured three times, and the average electrical resistance was calculated. Table 2 displays the electrical resistance values for the flexible thermoelectric module. The average electrical resistance for the 3.5 × 3.5 cm^2^ flexible thermoelectric module prototype obtained in this study was approximately 0.6 to 0.7 Ω. It is evident that the electrical resistance of each module is similar, indicating the consistency of the manufacturing process.

### 3.2. Experimental Results of FTEG Integrated with Flexible Heatsink

**Figure 5:**
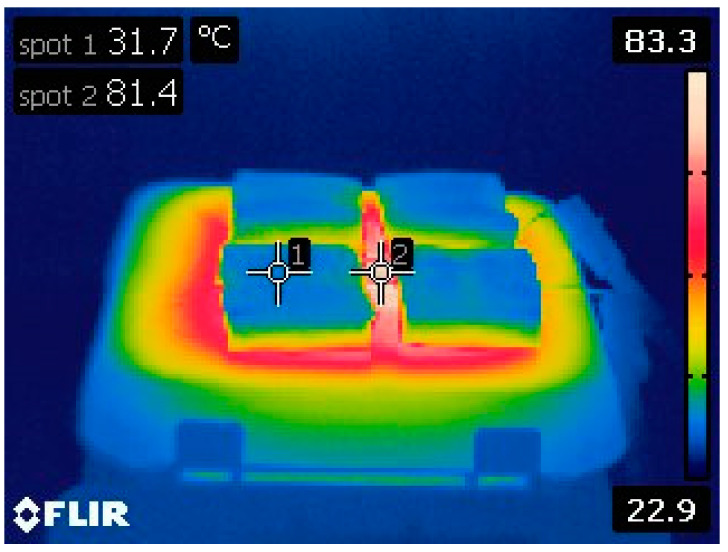
Experimental setup for monitoring phase change material leaks during operation.

To evaluate the efficacy of the phase-change material container in preventing leaks, the flexible phase-change material heatsink was placed on a hotplate, and its temperature was monitored using an IR (infrared) camera, as depicted in Figure 5. The hotplate’s temperature was precisely regulated at 80 °C. The operation was tested three times, revealing no instances of phase-change material leaks during the process. These results indicate the successful design and fabrication of the leak prevention container for the phase-change material during operation.

**Figure 6:**
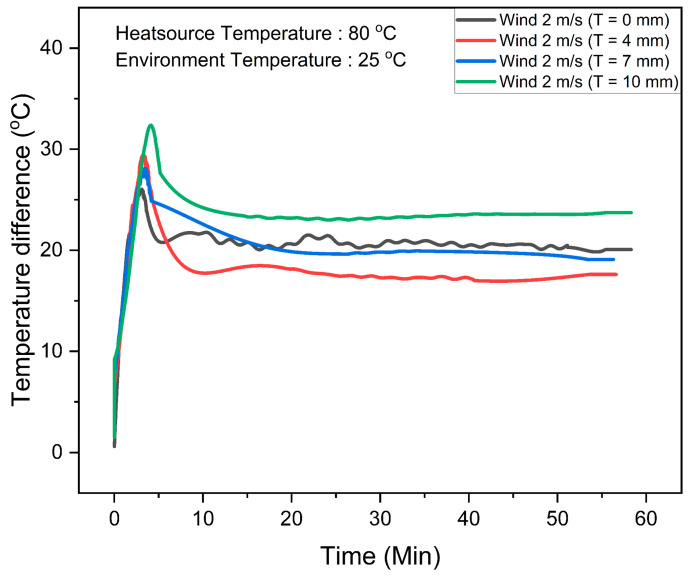
Temperature differences as functions of operating time for flexible thermoelectric generators, comparing those with and without flexible phase-change material heatsinks at a wind speed of 2 m/s.

To attain optimal performance from the FTEG and ensure an extended operational period, the heatsink must not only be flexible but also possess substantial heat capacity simultaneously. The total latent heat plays a crucial role in determining the retention time of the flexible phase-change material heatsinks; hence, increasing the quantity of PCM is necessary to prolong this retention period. To investigate this, we fabricated three heatsinks with varying amounts of PCM, each with heights of 4 mm, 7 mm, and 10 mm, respectively. The wind speed was set at 2 m/s, and in the experimental setup, K-type thermocouples were used to measure the temperatures on the hot side and cold side of FTEG. The temperatures of the hot side and the environment were kept steady at 80 °C and 25 °C, respectively. The temperature difference across the FTEG is depicted in Figure 6.

With the flexible phase-change material system installed alongside the FTEG, the temperature difference increases over time, peaking at approximately 5 min. Subsequently, the temperature difference gradually decreases. Integrating the FTEG with flexible phase-change material heatsinks at heights of 4 mm, 7 mm, and 10 mm resulted in maximum temperature differences of 27 °C, 28 °C, and 32 °C, respectively. In contrast, the FTEG without flexible phase-change material heatsinks reaches a maximum temperature difference of approximately 25 °C within the initial 5 min timeframe. After 5 min, the temperature differences in all cases decrease until reaching stability at 10 min. Notably, after this point, the temperature difference in the FTEG without flexible phase-change material heatsinks exceeded that of the FTEGs with 4 mm and 7 mm thicknesses of flexible phase-change material heatsinks. These findings suggest that integrating relatively low thickness of flexible phase-change material heatsinks with the FTEG enhances the temperature difference between its hot and cold sides within a specific time frame, leveraging the latent heat properties of the phase-change material. As the phase-change material absorbs more heat and reaches its melting point, the installed flexible phase-change material heatsinks impede the cooling of the FTEG on the cold side, subsequently reducing the temperature difference. In contrast, the FTEGs equipped with 10 mm thicknesses of flexible phase-change material heatsinks consistently maintained a higher temperature difference than those without flexible phase-change material heatsinks. These outcomes illustrate that the appropriate thickness of flexible phase-change material heatsinks, when coupled with wind speed considerations, has the potential to enhance the temperature difference in the FTEG.

**Figure 7:**
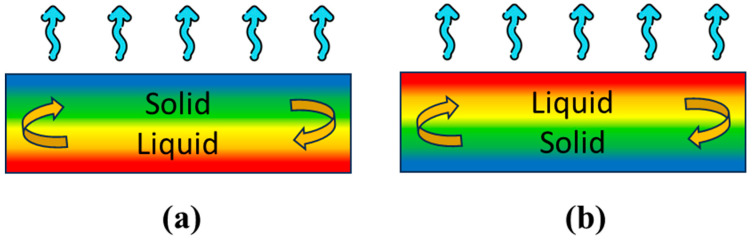
Illustrates the schematic model depicting the mechanism inside the phase change material when it absorbs heat from the FTEG. The arrows indicate phase transformation i.e. liquid to solid and solid to liquid.

When designing flexible phase-change material heatsinks, considering both melting and solidification behaviors becomes crucial. In passive PCM heatsinks, solidification often takes longer, posing challenges in handling [23]. Hence, ensuring adequate thickness and proper cooling for the flexible phase-change material heatsinks becomes essential to facilitate PCM solidification within a specified timeframe. However, a noticeable gap exists in the literature regarding the comprehension of flexible phase-change material heatsinks and the influence of wind speed convection during melting and solidification. This work addresses this gap by presenting a schematic model (Figure 7) that elucidates the effects of wind speed convection and PCM thickness throughout the melting and solidification processes.

To comprehend the heat dynamics within flexible phase-change material heatsinks, consider a scenario where both the temperature difference and maximum melting time increase with higher flexible phase-change material heatsink thicknesses. Once all phase changes have melted, heat convection occurs between the top area of the phase change material and the environment due to the applied wind speed. Consequently, this causes a decrease in the temperature of the top area of the phase change material, leading it to transform into the solid phase (Figure 7a). Subsequently, the high temperature of the liquid phase-change material at the bottom area moves towards the top area, while the solid phase-change material moves to the bottom area. As a result, the solid phase-change material absorbs heat from the flexible thermoelectric module, leading to an increased temperature difference (Figure 7b).

**Figure 8:**
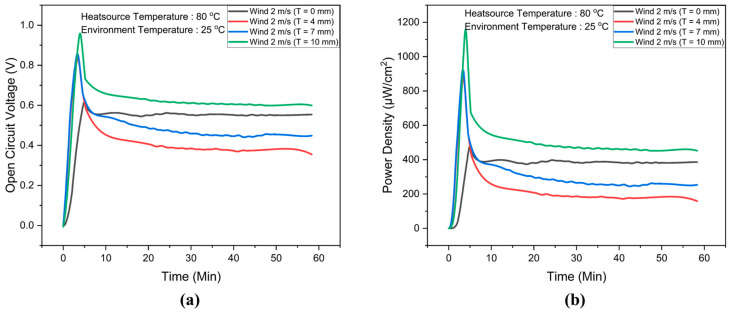
(**a**) Open-circuit voltage (*Voc*) and (**b**) power density data for the flexible thermoelectric system with and without flexible phase-change material heatsinks at a wind speed of 2 m/s, as a function of operating time.

The open-circuit voltage (*Voc*) data for the flexible thermoelectric system, both with and without flexible phase-change material heatsinks, are presented in Figure 8a. After 10 min, the FTEG integrated with flexible phase-change material heatsinks at a height of 10 mm exhibited an open-circuit voltage of 0.65 V, surpassing that of the FTEG without flexible phase-change material heatsinks. In contrast, the maximum open-circuit voltage observed for FTEGs with flexible phase-change material heatsinks at heights of 4 mm and 7 mm was lower than that of the FTEG without flexible phase-change material heatsinks. The relationships between power density and temperature difference are illustrated in Figure 8b. After 10 min, the FTEG equipped with a flexible phase-change material heatsink at a height of 10 mm achieved the highest power density, reaching 500 µW/cm². These experimental results underscore the critical need for a flexible and bendable heat dissipation system when integrated with the FTEG to generate adequate electrical power for practical applications, such as powering small electronic devices.

**Figure 9:**
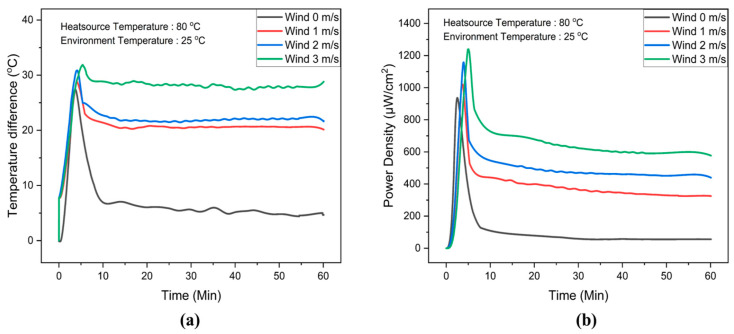
(**a**) Temperature differences and (**b**) power density data for the flexible thermoelectric integrated with flexible phase-change material heatsinks, comparing a 10 mm thickness with and without wind speed, across different operating times.

To investigate the influence of wind speed on the temperature difference and power density output of the FTEG integrated with a flexible phase-change material heatsink, wind speeds of 0, 1, 2, and 3 m/s were generated. The ambient temperature (25 °C) was controlled using an air conditioner. A flexible phase-change material heatsink with a thickness of 10 mm was integrated with the FTEG. The temperature difference and power density data are presented in Figure 9.

Figure 9a presents the temperature difference across the FTEG at various wind speeds. The temperature difference increases with higher wind speeds. The average temperature differences were 5 °C, 20 °C, 21 °C, and 30 °C corresponding to wind speeds of 0 m/s, 1 m/s, 2 m/s, and 3 m/s, respectively. Furthermore, the temperature difference across the FTEG remains almost stable at a wind speed of 3 m/s. The power density is shown in Figure 9b7b: a power density of 100 µW/cm² is obtained when the FTEG is integrated with a flexible phase-change material heatsink without any applied wind in the system. It is noticeable that the power density achieved is more than four times higher when the wind speed is at 1 m/s compared to no wind speed. This result indicates that integrating phase-change material heatsinks and relatively low wind speeds can significantly enhance flexible thermoelectric generator efficiency. In addition, the FTEG equipped with a 10 mm thick flexible phase-change material heatsink, combined with a 3 m/s wind speed, achieved the highest power density, reaching 1250 µW/cm^2^. Moreover, this integrated system sustained a power output exceeding 625 µW/cm^2^ for 60 min.

Table 3 presents the performance of FTEG in previous studies and this study. Our developed FTEG, alongside flexible phase-change material heatsinks with and without wind speed, exhibits higher output power and greater power density than in recent publications [14,16,17,18,24]. In our understanding, this figure is notably elevated when contrasted with other documented FTEG measurements, showcasing the efficacy of our design and the prospective usefulness of the FTEG.

### 3.3. Application Demonstrations

**Figure 10:**
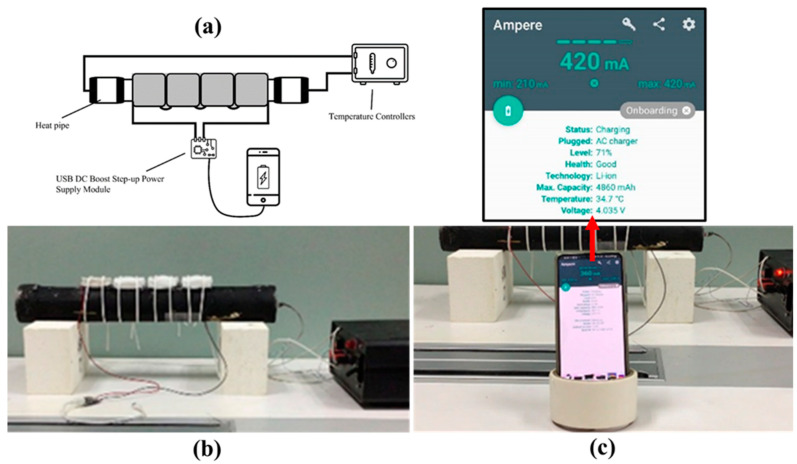
(**a**) The schematic of the smartphone charging demonstration setup, (**b**) four flexible thermoelectric generators were affixed to the hot pipe at a temperature setup of 80 °C, and (**c**) the smartphone was charged and monitored using the Ampere application.

To demonstrate the potential of the constructed FTEG integrated with the flexible phase-change material heatsink and relatively low wind speed for energy harvesting, an experimental setup was utilized, as depicted in Figure 10a. Four flexible thermoelectric modules were connected in a series, and they were covered with a graphite sheet. Thermal grease was used to reduce the thermal resistance at the contact points. The four flexible thermoelectric generators were affixed to the hot pipe, and the flexible heatsink was employed (Figure 10b). In this experiment, a wind speed of 1 m/s was used. The USB DC boost step-up module is connected to the smartphone, and the charging module, in turn, is connected to flexible thermoelectric generators. As the temperature difference across the FTEG materialized, electricity was generated at the output terminal of the FTEG. To elevate a low voltage to a higher level, a step-up DC-to-DC converter is commonly employed in low-voltage harvesting. This converter achieves a higher voltage by reducing the current, enabling power preservation and attainment of the desired voltage level. In this study, step-up DC-to-DC converters are utilized, given the low-power harvesting context. With the harvested low voltage, the goal is to power a mobile device. The smartphone charging was monitored using the Ampere application as presented in Figure 10c.

## 4. Conclusions

This research introduces an innovative and groundbreaking concept for a flexible heatsink by integrating a phase-change material. Three different thicknesses (4 mm, 7 mm, and 10 mm) were created and used to regulate the heat dissipation of the flexible thermoelectric device. The design of the flexible phase-change material heatsink was specifically engineered to prevent leaks during operation. When the FTEG incorporated flexible phase-change material heatsinks at heights of 4 mm, 7 mm, and 10 mm, it exhibited peak temperature differences of 27 °C, 28 °C, and 32 °C, respectively. After 10 min of operation, the temperature difference in the FTEG without flexible phase-change material heatsinks surpassed that of the FTEGs with 4 mm and 7 mm thicknesses of flexible phase-change material heatsinks. Conversely, FTEGs equipped with 10 mm thicknesses of flexible phase-change material heatsinks consistently maintained higher temperature differences than those without flexible phase-change material heatsinks. Moreover, the FTEG equipped with a 10 mm thick flexible phase-change material heatsink, combined with relatively low wind speed, significantly enhanced the power density. Finally, we demonstrated a real-world application using the developed flexible thermoelectric generator combined with the FPCM heatsink, effectively converting excess heat from a circular hot pipe into electrical energy to power smartphones and similar devices.

## Figures and Tables

**Table 1:** Thermophysical properties of RT42 [24].

Properties	Unit	RT42
Solidus temperature	°C	38
Liquidus temperature	°C	42
Solid density	kg/m^3^	880
Liquid density	kg/m^3^	760
Specific heat	J/(kg·K)	2000
Latent heat	J/kg	165,000
Thermal expansion coefficient	1/K	0.0001
Thermal conductivity	W/(m·K)	0.2

**Table 2:** The resistance and average resistance of the four prototype flexible thermoelectric modules.

Flexible Thermoelectric	Resistance (Ω)				S.D
	R_1_	R_2_	R_3_	R_averge_	
A	0.60	0.70	0.70	0.67	0.06
B	0.60	0.70	0.60	0.63	0.06
C	0.70	0.70	0.80	0.73	0.06
D	0.80	0.60	0.70	0.70	0.10

**Table 3:** Comparison of FTEG performance.

Heat Sink	Wind Speed (m/s)	Power Density (µW/cm^2^)	Reference
Copper electrodes	2	48	[14]
Copper foam	-	15	[16]
Assembled copper foam onto PDMS film	-	16	[17]
Assembled copper foam onto PDMS film	0.8	98	[17]
Phase-change material (CaCl_2_·6H_2_O)	-	35	[25]
Phase-change inorganics (CaCl_2_·6H_2_O)	1.5	50	[25]
Heteromorphic copper electrode	-	21	[18]
Heteromorphic copper electrode	2.1	116	[18]
Phase-change material (RT42)	-	100	This work
Phase-change material (RT42)	1	400	This work
Phase-change material (RT42)	3	625	This work

## Data Availability

All data in this manuscript used to support the findings of this study are available from the corresponding author upon request.
